# Genome Sequence of Bacteriophages Bouchard and Windest Isolated Using *Arthrobacter globiformis* B-2979

**DOI:** 10.17912/micropub.biology.001885

**Published:** 2026-02-11

**Authors:** Taryn Marcum, Corena Racine, Kyle Johnson, Tyler Hildebrand, John Patton

**Affiliations:** 1 Natural and Applied Sciences, Evangel University, Springfield, Missouri, United States

## Abstract

Bacteriophages Bouchard and Windest are siphoviruses isolated using *Arthrobacter globiformis* B-2979-SEA. The genome of Bouchard is predicted to encode 91 proteins within its 57,304 bp genome, whereas the genome of Windest is predicted to encode 99 proteins and two tRNAs within its 51,773 bp genome. Based on gene content similarity of at least 35% to actinobacteriophages, Bouchard and Windest have been assigned to the AU2 subcluster and AY cluster of Actinobacteriophages, respectively.

**Figure 1. Plaque and virion morphology:**
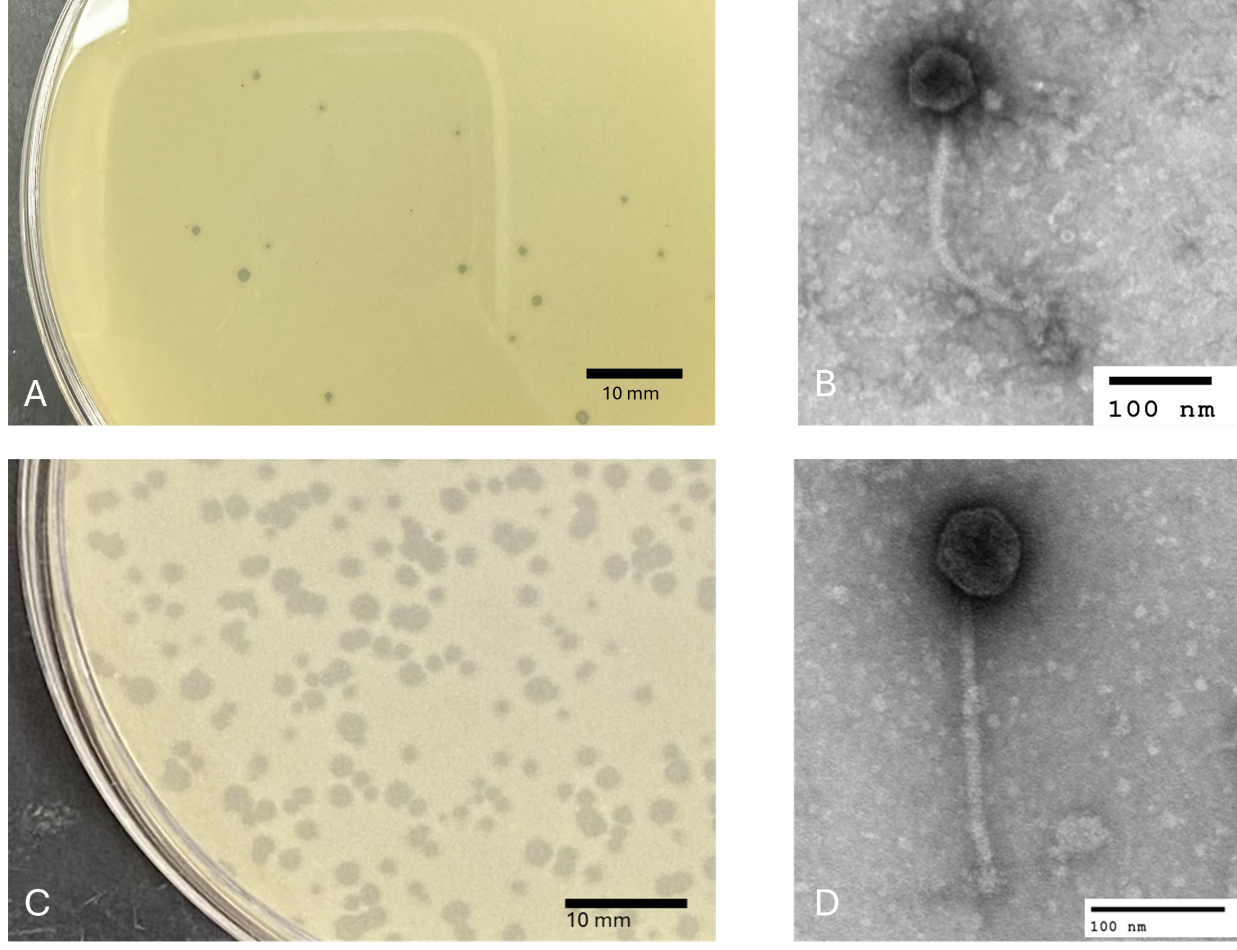
A. Bouchard generates clear plaques with well-defined borders. B. Bouchard particles have siphovirus morphology with a polyhedral capsid and long, flexible tail. C. Windest generates clear plaques with well-defined borders. D. Windest particles have siphovirus morphology with a polyhedral capsid and long, flexible tail.

## Description

**Description**

*Arthrobacter* species have potential uses in degrading hazardous organic compounds (Sahoo et al., 2010) and reducing chromium(VI) (Silva et al., 2012). Advancing our understanding of *Arthrobacter* species and the bacteriophages that infect them will increase the potential for application. Here, we report the genomes of two bacteriophages, Bouchard and Windest, that infect *Arthrobacter globiformis* B-2979-SEA.

Bouchard was isolated from a soil sample collected in Branson, MO, USA (GPS: 36.66271 N, 93.26668 W). Windest was isolated from a soil sample collected in a drainage ditch in El Dorado Springs, MO, USA (GPS: 37.85757 N, 94.01873 W). Each sample was incubated with peptone-yeast-calcium (PYCa) liquid medium for two hours with shaking at 250 rpm. The suspension was then centrifuged at 2000 x g for 10 minutes, and the supernatant was filtered (0.22 µm pore size). The filtrate was then inoculated with *A. globifomis* B-2979-SEA and incubated at 30°C for three days with shaking. The resulting culture was filtered to remove the bacteria (0.22 µm pore size), and the filtrate was plated in PYCa top agar with *A. globiformis* , producing plaques within two days of incubation at 30°C. One plaque for each soil sample was picked and purified through three rounds of plating (Zorawik et al., 2024). Bouchard produced plaques with a halo morphology and irregular borders with a diameter of 1.6 mm ± 0.1 mm (n=5). Windest produced clear, circular plaques with a diameter of 1.4 mm ± 0.2 mm (n=5) (Figure 1). A high titer lysate was prepared for each phage, and transmission electron microscopy was performed on the lysate using negative staining with 1% uranyl acetate. Each bacteriophage was shown to have a siphovirus morphology. Bouchard has a capsid diameter of 65.6 ± 6.5 nm (n=5) and a tail length of 236.7 ± 31.5 nm (n=5). Windest has a capsid diameter of 63.0 ± 5.9 nm (n=5) and a tail length of 217.2 ± 12.2 nm (n=5) (Figure 1).

DNA was isolated from the high titer lysates of Bouchard and Windest with the Promega Wizard DNA Clean-Up kit. The genomes were then sequenced with an Illumina NextSeq 1000 with the XLEAP-P1 kit after library preparation with a NEB Ultra II FS Library kit. Raw reads were trimmed with cutadapt 4.7 (using the option: –nextseq-trim 30) and filtered with skewer 0.2.2 (using the options: -q 20 -Q 30 -n -l 50) prior to assembly. Sequencing the genome of Bouchard yielded 5,040,960, 100-bp reads, which were assembled into a genome with 8,468-fold coverage using Newbler v2.9 (Miller et. al. 2010). The resulting genome was 57,304 bp in length with a GC content of 50.4% and 3′ single-stranded overhangs (9 bases, sequence: 5'-CGCCGGCCC-3') determined by Consed v29 (Gordon and Green, 2013) following Russell (2018). The genome of Windest yielded 4,672,765, 100-bp reads, which was assembled with 8,691-fold coverage. The resulting genome was 51,773 bp in length with a GC content of 62.6% and 3′ single-stranded overhangs (9 bases, sequence: 5'-CGCCGGTGA-3') determined by Consed v29 (Gordon and Green, 2013) following Russell (2018).

All annotations were performed using PECAAN v20250130 (Rinehart et. al., 2016) before importing into DNA Master v5.23.6 for formatting into a file for GenBank submission (Pope and Jacobs-Sera, 2018). Possible start sites for translation were identified using GeneMark v2.5 (Besemer and Borodovsky, 2005) and Glimmer v3.02 (Delcher et al., 2007). These translational start sites were then refined using Starterator (http://phages.wustl.edu/starterator/) and Phamerator, using the Actino_draft database v 578 (Cresawn et al., 2011) to compare conserved start sites in other previously annotated genomes. Gene functions were assigned on PECAAN (Rinehart et al., 2016) utilizing the integrated BLAST searches against the Actinobacteriophage and NCBI non-redundant databases (Altschul et al., 1990) searches against the NCBI non-redundant and Actinobacteriophage databases (Russell and Hatfull, 2017) and HHPred searches against the PDB_mmCIF70, SCOPe70 _2.08, and NCBI_Concerved_Domains (CD) v3.19 databases (Söding et al., 2005). Both genomes were analyzed for potential tRNAs utilizing tRNA scan v2.0 (Lowe and Eddy, 1997) and ARAGORN v1.2.41 (Laslett and Canback, 2004). DeepTMHMM v1.0 (Hallgren et al., 2022) was utilized to identify putative membrane proteins. Based on gene content similarity of at least 35% when compared with phages in the Actinobacteriophage database, phages Bouchard and Windest were assigned to clusters AU and AY, respectively (Pope et al., 2017; Russell and Hatfull, 2017). Default settings were used for all software tools.

Bouchard has 91 predicted protein-coding genes, 25 of which could be assigned a putative function. Windest has 99 predicted protein-coding genes, 44 of which could be assigned a putative function. Bouchard and Windest have 10 and 6 predicated membrane proteins, respectively. Bouchard lacks tRNA genes, whereas Windest has two tRNA genes: one with an anticodon for tryptophan and the other with an undetermined anticodon assignment. Consistent with other subcluster AU2 phages, Bouchard lacks identifiable integrase and immunity repressor genes, suggesting these phages are unlikely to establish lysogeny. Windest, on the other hand, encodes a predicted tyrosine integrase gene that is conserved across all cluster AY phages. Although no immunity repressor has been identified for any phages of this cluster, they are predicted to be temperate. Lysogens for at least one cluster AY phages has been raised (Hussain et. al., 2025).

**Nucleotide Sequence Accession Numbers:**

Bouchard is available at GenBank with Accession Number PV876923 and Sequence Read Archive Number SRX28943188 .

Windest is available at GenBank with Accession Number PV876971 and Sequence Read Archive Number SRX28943187 .
